# Inhibitory Activities of *Cudrania tricuspidata* Leaves on Pancreatic Lipase *In Vitro* and Lipolysis *In Vivo*

**DOI:** 10.1155/2012/878365

**Published:** 2012-12-10

**Authors:** Young Sook Kim, Youngseop Lee, Junghyun Kim, Eunjin Sohn, Chan Sik Kim, Yun Mi Lee, Kyuhyung Jo, Sodam Shin, Yoojin Song, Joo Hwan Kim, Jin Sook Kim

**Affiliations:** ^1^Korean Medicine-Based Herbal Drug Research Group, Herbal Medicine Research Division, Korea Institute of Oriental Medicine (KIOM), Daejeon 305-811, Republic of Korea; ^2^Department of Life Science, Gachon University, Seongnam, Kyonggi-do 461-701, Republic of Korea

## Abstract

To identify effective herb to treat obesity, we screened 115 herbal extracts for inhibition of porcine pancreatic lipase (triacylg-ycerol acylhydrolase, EC 3.1.1.3) activity *in vitro*. Of the extracts tested, *Cudrania tricuspidata* leaves exhibited the most pronounced inhibitory effect on lipase activity with an IC_50_ value of 9.91 **μ**g/mL. Antilipid absorption effects of *C. tricuspidata* leaves were examined in rats after oral administration of lipid emulsions containing 50 or 250 mg  *C. tricuspidata*/kg body weight. Plasma triacylglycerol levels 2 h after the oral administration of emulsions containing *C. tricuspidata* were significantly reduced compared to the untreated group (*P* < 0.05). These results suggest that *C. tricuspidata* leaves may be useful for the treatment of obesity.

## 1. Introduction

Obesity is a significant risk factor for increased morbidity and mortality from cardiovascular disease and diabetes; however, it is also associated with many other medical conditions including cancer, liver and kidney diseases, sleep apnea, and depression [1]. The recent National Health and Nutrition Examination Survey showed that 68.0% of those studied were considered overweight (basal metabolic rate (BMI) ≥ 25) and 33.8% were obese (BMI ≥ 30) [2]. The inhibition of dietary fat absorption is a logical target for managing obesity, and pancreatic lipase is a key enzyme involved in triglyceride absorption in the small intestine. It is secreted from the pancreas and hydrolyzes triglycerides into glycerol and free fatty acids. Thus, inhibitors of digestive lipases are suggested to function as antiobesity agents [3]. Orlistat, which can be found in global markets, inhibits the action of gastrointestinal lipase and thus reduces absorption of dietary fat. However, it has serious side effects, such as steatorrhea, stomach pain, irregular menstrual periods, and headaches [4]. Recently, studies have searched for new lipase inhibitors in natural resources with minimal adverse effects. In a series of investigations to evaluate potential lipase inhibitors derived from plants, researchers showed that certain plant extracts significantly inhibited porcine pancreatic lipase *in vitro* [5, 6]. In this study, as a preliminary evaluation of natural antiobesity products, we tested 115 herbal extracts for inhibition of pancreatic lipase activity *in vitro* and verified the suppression of lipid absorption by *C. tricuspidata* leaves *in vivo*. The fruits of *C. tricuspidata *suppress development of atopic dermatitis in animal model and the roots of it exhibit immunomodulatory and anti-oxidant activities *in vitro* [7, 8]. These results show that *C. tricuspidata *leaves extracts have on lipase and dietary fat absorptionactivities and may be useful in the treatment of obesity and metabolic disease.

## 2. Material and Methods

### 2.1. Plant Materials and Chemicals

Herbs were collected from Republic of Korea from September 2005 to July 2009 and identified by Professor Kim, Division of Life Science, Gachon University, Republic of Korea. Samples were deposited at the Herbarium of Diabetic Complication Research Team, Korea Institute of Oriental Medicine. Porcine pancreatic lipase (type II), orlistat, and *p*-nitrophenyl butyrate were purchased from Sigma-Aldrich (St. Louis, MO, USA). All reagents were of biochemical grade.

### 2.2. Animals

Male Wistar rats (6 weeks of age) were purchased from Koatech (Kyungkido, Korea) and housed for 1 week in a 12-h/12-h light/dark cycle in a temperature- and humidity-controlled room. The animals were given free access to food and water. After adaptation to these conditions for 1 week, healthy animals were used in the present study. The Animal Studies Committee of Korea Institute of Orient Medicine approved the experimental protocol.

### 2.3. Preparation of Herbal Extracts

Dried and ground herbs (200 g) were extracted with 1 L of 80% EtOH 3 times by maceration. The extracts were concentrated and dried *in vacuo* at 40°C. Concentrated extracts were stored at −20°C for further studies. Extracts were dissolved in dimethyl sulfoxide at concentrations that in the total volume (3%) did not affect enzyme activity.

### 2.4. Measurement of Porcine Pancreatic Lipase Inhibitory Activity

The ability of the herbs to inhibit pancreatic lipase was measured using the method previously reported by Kim et al. [9, 10]. Briefly, an enzyme buffer was prepared by the addition of 6 *μ*L porcine pancreatic lipase solution (Sigma-Aldrich) in buffer containing 10 mM MOPS (morpholinepropanesulphonic acid) and 1 mM EDTA, pH 6.8, to 169 *μ*L Tris buffer (100 mM Tris-HC1 and 5 mM CaCl_2_, pH 7.0). Then, 20 *μ*L of either the herbal extracts at the test concentration (0, 0.313, 0.625, 1.25, 2.5, 5, 7.5, 10, 50, and 100 *μ*g/mL) or orlistat (Roche, Basel, Switzerland) were mixed with 175 *μ*L enzyme buffer and incubated for 15 min at 37°C with 5 *μ*L substrate solution (10 mM *p*-NPB (*p*-nitrophenylbutyrate) in dimethyl formamide); the enzymatic reactions were allowed to proceed for 15 min at 37°C. Lipase activity was determined by measuring the hydrolysis of *p*-NPB to *p*-nitrophenol at 405 nm using an ELISA reader (BIO-TEK, Synergy HT, Winooski, VT, USA). Inhibition of lipase activity was expressed as the percentage decrease in OD when porcine pancreatic lipase was incubated with the test materials. Lipase inhibition (%) was calculated according the following formula:

(1) $$\begin{array}{c} \text{Inhibition}\;\;\left(\%\right)=100-\left(\frac{B-b}{A-a}\times 100\right), \end{array}$$

where *A* is the activity without inhibitor, *a* is the negative control without inhibitor, *B* is the activity with inhibitor, and *b* is the negative control with inhibitor. The results were expressed as an average (*n* = 3).

### 2.5. Estimation of Plasma Triacylglycerol after Oral Administration of Lipid Emulsion in Rats

Plasma triacylglycerol levels were estimated using the method previously reported by Kim et al. [11]. Rats (7 weeks of age, body weight 190 ~ 230 g) that had fasted overnight were orally administered 3 mL lipid emulsion consisting of corn oil (6 mL), cholic acid (80 mg), cholesteryloleate (2 g), and saline (6 mL) with or without *C. tricuspidata* leaves (at doses of 50 or 250 mg *C. tricuspidata* leaves/kg body weight). Blood was taken from the tail vein at 0, 1, 2, 3, and 4 h after oral administration of the lipid emulsion and centrifuged at 5500 ×g for 5 min to obtain the plasma. Triacylglycerol levels were determined using the Cleantech TS-s kit (ASANPHARM, Seoul, Korea).

### 2.6. Statistical Analysis

All experiments were repeated three times, and representative data are shown. Data are expressed as the mean ± S.D. Differences between groups were analyzed using a one-way ANOVA followed by the Tukey multiple comparison test (PRISM software, Graph Pad, CA, USA). Values of *P* < 0.05 were considered statistically significant. 

## 3. Results and Discussion

### 3.1. Pancreatic Lipase Activity of Herbal Extracts

**Figure 1:**
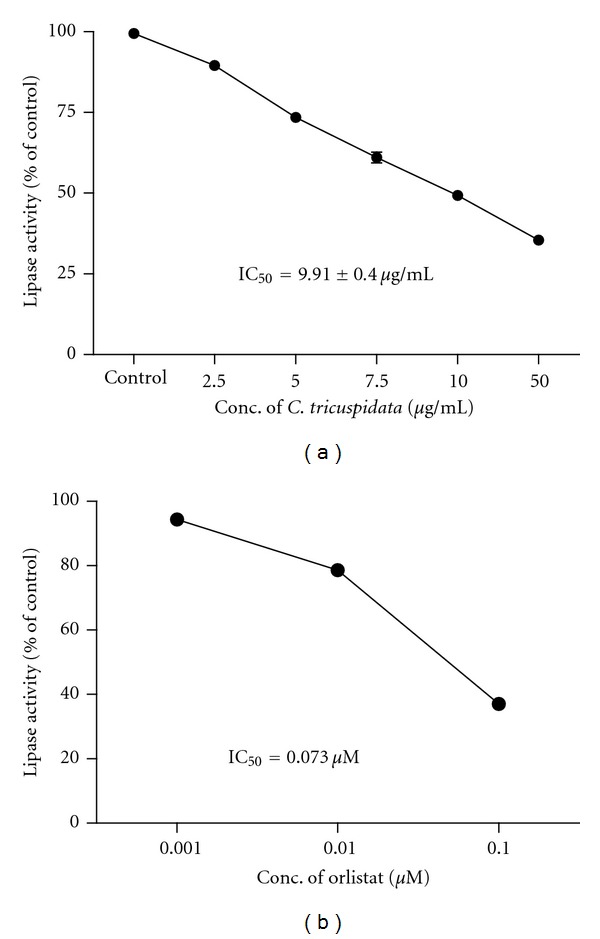
Inhibitory effect of *Cudrania tricuspidata *leaf extract on porcine pancreatic lipase. (a) Porcine pancreatic lipase activity at different concentrations of *C. tricuspidata *leaves. (b) Orlistat was used as a positive control. Data are the mean ± S.D. (*n* = 3).

Currently, obesity is considered a global epidemic, and many medications have been studied and developed to treat this condition. However, there is presently only one drug—orlistat—globally approved for long-term treatment of overweight patients after sibutramine was withdrawn in January 2010 from the European market [12, 13]. Although this compound strongly inhibits the activity of pancreatic lipase, which is an important enzyme associated with fat digestion, orlistat may cause serious adverse effects on the gastrointestinal, nervous, endocrine, and renal systems and interferes with the absorption and effectiveness of many drugs and vitamins [4, 14]. Therefore, researching a safe and effective natural inhibitor of pancreatic lipase has been a major target for the development of new drugs to treat obesity [15]. Among them, extracts isolated from natural sources such as *Sorbus commixta*,* Morus bombycis*,* Panax ginseng*, and *Ginkgo biloba* have been reported as potential agents in pancreatic lipase inhibition action [16–19]. Our previous studies have also identified some natural products as new pancreatic lipase inhibitors [11, 18, 19]. In this study, 115 herbal extracts were prepared from selected parts of plants and tested at various concentrations as inhibitors of pancreatic lipase. The lipase inhibitory effects of the extracts are indicated by percentage (%) and IC_50_ values (Table 1). Eighteen extracts had IC_50_ values less than 50 *μ*g/mL, and of these extracts, three samples (i.e., the whole *Solidago serotina *plant, the branches and leaves of *Acer mono*, and the leaves of *C. tricuspidata*) had IC_50_ values less than 10 *μ*g/mL. Notably, *C. tricuspidata* leaves exhibited an IC_50_ value of 9.91 *μ*g/mL (Figure 1).

### 3.2. Inhibitory Effect of *C. tricuspidata* on Lipolysis *In Vivo*

**Figure 2:**
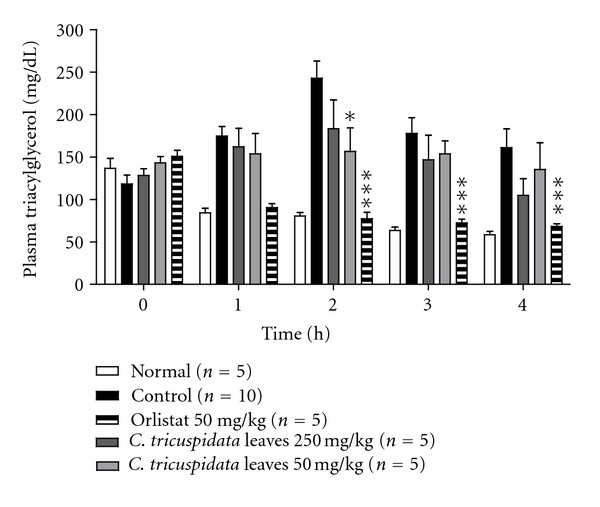
Inhibitory effect of *Cudrania tricuspidata* leaves on rat plasma triacylglycerol levels. Plasma triacylglycerol levels, at the time marked by an asterisk, significantly differ between the control and *C. tricuspidata* (250 mg/kg) groups (*P* < 0.05). Orlistat (a lipase inhibitor) was used as a positive control (*P* < 0.001 versus control).

Next, we focused on *C. tricuspidata *on lipolysis* in vivo*. *C. tricuspidata *has been used as an important folk medicine for the treatment of cancer in Korea and has also been used as a traditional medicine for the treatment of hypertension, neuritis, and inflammation in Asia [20–22]. To evaluate the antilipolytic effects of *C. tricuspidata* leaves *in vivo*, we analyzed plasma triacylglycerol levels after oral administration of lipid emulsions with or without the *C. tricuspidata *leaves to rats.  Figure 2 shows plasma triacylglycerol levels after oral administration of lipid emulsion with or without *C. tricuspidata *as a function of time. After oral administration, low concentrations of *C. tricuspidata* (50 mg/kg body weight) reduced plasma triacylglycerol levels and high concentrations of *C. tricuspidata* (250 mg/kg body weight) delayed lipid absorption significantly; however, these effects were weaker than that of the positive control, orlistat.

*C. tricuspidata* is a rich source of xanthones and flavonoids, including cudraflavone C [23]. A recent study reported that cudraflavone C from *Artocarpus nitidus* inhibited pancreatic lipase activity (IC_50_ = 17.0 ± 0.7 *μ*M) [24]. Thus, cudraflavone C may be a potential as one of active compounds for preventing and treating obesity.

## 4. Conclusion

In this paper, we screened 115 herbal extracts for inhibition of porcine pancreatic lipase to identify effective herb to treat obesity. *C. tricuspidata *leaves show the most pronounced effect on pancreatic lipase activity and are able to suppress dietary fat absorption *in vivo*. Up until now, *C. tricuspidata *leaves extracts have not been reported on lipase and dietary fat absorptionactivities. Thus, it is worthwhile to further investigate these extracts for their potential pharmacological effect in antiobesity and attempt should be made to characterize phytoactive compounds to be used as safer therapeutic agents in future.

## Figures and Tables

**Table 1:** Lipase inhibitory activities of extracts from herbs.

Scientific name	Family	Part used	Conc. (*μ*g/mL)	Inhibition (%)^a^	IC_50_ (*μ*g/mL)
			2.5	41.76 ± 2.48	
*Solidago serotina *	Compositae	Whole plant	5	49.70 ± 1.44	5.16
			7.5	55.70 ± 1.81	
			5	46.17 ± 3.03	
*Acer mono *	Aceraceae	Branch, leaf	7.5	48.87 ± 3.09	7.7
			10	53.16 ± 0.93	
			5	26.55 ± 0.52	
*Cudrania tricuspidata *	Moraceae	Leaf	7.5	38.97 ± 2.92	9.91
			10	50.72 ± 1.05	
			10	49.77 ± 1.00	
*Kalopanax pictus *	Araliaceae	Bark	50	70.52 ± 1.70	10.51
			100	76.34 ± 0.36	
			5	32.34 ± 2.04	
*Cudrania tricuspidata *	Moraceae	Branch, stem	10	48.29 ± 1.19	13.8
			50	65.83 ± 0.29	
			10	45.06 ± 1.81	
*Oenothera odorata *	Onagraceae	Whole plant	50	59.58 ± 0.70	23.34
			100	61.07 ± 0.63	
			10	45.08 ± 4.01	
*Platycarya strobilacea *	Juglandaceae	Branch, stem	50	56.72 ± 1.74	25.51
			100	61.74 ± 1.26	
			10	41.62 ± 7.54	
*Actinidia arguta *	Actinidiaceae	Fruit	50	59.30 ± 0.80	26.7
			100	67.23 ± 3.20	
			10	41.72 ± 2.86	
*Tilia amurensis *	Tiliaceae	Branch, leaf	50	59.26 ± 0.55	28.5
			100	67.17 ± 1.03	
			10	36.79 ± 0.82	
*Actinidia arguta *	Actinidiaceae	Stem	50	63.38 ± 2.42	28.51
			100	66.84 ± 2.70	
			20	43.12 ± 4.05	
*Euscaphis japonica *	Staphyleaceae	Branch	30	50.91 ± 1.29	28.62
			40	56.29 ± 2.10	
			10	34.08 ± 1.94	
*Actinidia arguta *	Actinidiaceae	Root	50	63.93 ± 1.94	31.34
			100	71.03 ± 0.89	
			10	44.19 ± 3.68	
*Carpinus cordata *	Betulaceae	Branch, stem	50	54.25 ± 1.11	31.39
			100	58.91 ± 1.62	
			10	41.57 ± 2.64	
*Rhus sylvestris *	Anacardiaceae	Branch, leaf	50	57.23 ± 4.33	32.14
			100	57.43 ± 2.28	
			10	41.52 ± 1.71	
*Celtis sinensis *	Ulmaceae	Branch, stem	50	54.56 ± 0.52	35.89
			100	54.09 ± 3.37	
			10	34.40 ± 2.70	
*Prunus serrulata *	Rosaceae	Branch, leaf	50	53.53 ± 0.62	42.55
			100	56.43 ± 3.18	
			10	28.48 ± 4.40	
*Potentilla fragarioides *	Rosaceae	Whole plant	50	54.81 ± 2.36	42.58
			100	61.88 ± 1.34	
			10	32.90 ± 4.37	
*Tilia mandshurica *	Tiliaceae	Flower, leaf	50	51.59 ± 2.07	48.21
			100	52.74 ± 2.30	
			10	19.86 ± 2.15	
*Actinidia arguta *	Actinidiaceae	Stem, leaf, fruit	50	50.25 ± 2.65	54.09
			100	56.92 ± 2.15	
			10	28.85 ± 6.19	
*Hypericum ascyron *	Hypericaceae	Whole plant	50	49.57 ± 5.42	56.12
			100	57.57 ± 3.13	
			10	37.15 ± 0.50	
*Rhus chinensis *	Anacardiaceae	Branch, leaf	50	49.65 ± 0.66	56.9
			100	52.06 ± 1.66	
			10	23.97 ± 2.01	
*Picrasma quassioides *	Simaroubaceae	Branch, stem	50	48.78 ± 0.80	60.47
			100	54.89 ± 1.38	
			10	26.90 ± 1.18	
*Prunus persica *	Rosaceae	Branch, leaf	50	48.04 ± 0.94	62.12
			100	56.27 ± 1.46	
			10	12.22 ± 5.84	
*Actinidia arguta *	Actinidiaceae	Root	50	45.58 ± 3.38	69.17
			100	56.48 ± 1.93	
			10	24.96 ± 2.54	
*Spiraea pubescens *	Rosaceae	Branch, leaf, flower	50	47.25 ± 3.35	74.62
			100	52.19 ± 1.37	
			10	17.77 ± 3.99	
*Tilia mandshurica *	Tiliaceae	Branch, stem	50	44.39 ± 2.14	79.67
			100	54.07 ± 2.85	
			10	17.93 ± 2.59	
*Acer ginnala *	Aceraceae	Branch, leaf	50	43.30 ± 3.02	82.29
			100	53.89 ± 2.92	
			10	20.95 ± 3.37	
*Elsholtzia splendens *	Labiatae	Root	50	44.64 ± 1.74	83.98
			100	52.58 ± 1.67	
			10	28.75 ± 5.25	
*Staphylea bumalda *	Staphyleaceae	Branch, leaf	50	42.55 ± 2.40	84.28
			100	53.45 ± 2.55	
			80	49.17 ± 1.04	
*Pinus densiflora *	Pinaceae	Stem	90	49.77 ± 3.57	87.58
			100	52.63 ± 2.09	
			10	29.96 ± 8.94	
*Machilus thunbergii *	Lauraceae	Leaf, branch	50	45.82 ± 0.31	90.9
			100	50.93 ± 0.00	
			10	27.34 ± 8.43	
*Deutzia glabrata *	Saxifragaceae	Branch, leaf, flower	50	42.85 ± 2.09	91.09
			100	51.51 ± 1.46	
			10	22.19 ± 1.39	
*Indigofera kirilowii *	Leguminosae	Branch, leaf, flower	50	39.83 ± 0.73	94.98
			100	51.24 ± 1.32	
*Opuntia ficus-indica *	Opuntiacae	Stem	100	28.17 ± 1.66	>100
*Hibiscus syriacus *	Malvaceae	Root	100	13.95 ± 0.72	>100
*Actinidia arguta *	Actinidiaceae	Bark	100	26.02 ± 8.63	>100
*Euonymus oxyphyllus *	Celastraceae	Branch	100	47.50 ± 0.76	>100
*Eucommia ulmoides *	Eucommiaceae	Branch, leaf	100	37.76 ± 0.89	>100
*Asarum sieboldii *	Aristolochiac	Root	100	15.50 ± 5.18	>100
*Bupleurum longeradiatum *	Umbelliferae	Whole plant	100	34.69 ± 2.52	>100
*Plantago asiatica *	Plantaginacea	Root	100	−14.66 ± 4.59	>100
*Alisma plantago-aquatica *	Alismataceae	Root	100	22.03 ± 4.65	>100
*Duchesnea chrysantha *	Rosaceae	Whole plant	100	36.69 ± 1.07	>100
*Cuscuta japonica *	Convolvulaceae	Whole plant	100	2.43 ± 1.75	>100
*Clematis apiifolia *	Ranunculaceae	Stem, leaf, flower	100	−19.96 ± 1.10	>100
*Prunus serrulata *	Rosaceae	Branch	100	43.47 ± 0.18	>100
*Colocasia antiquorum *	Araceae	Aerial part	100	−12.08 ± 3.87	>100
*Lespedeza cuneata *	Leguminosae	Aerial part	100	−8.62 ± 2.65	>100
*Lespedeza cuneata *	Leguminosae	Root	100	−4.14 ± 1.86	>100
*Mallotus japonicas *	Euphorbiaceae	Aerial part	100	11.45 ± 3.84	>100
*Alisma canaliculatum *	Alismataceae	Aerial part	100	16.36 ± 2.85	>100
*Alisma canaliculatum *	Alismataceae	Root	100	26.99 ± 0.41	>100
*Magnolia denudata *	Magnoliaceae	Flowers	100	−5.01 ± 2.23	>100
*Scopolia japonica *	Solanaceae	Stem, leaf	100	−10.52 ± 0.76	>100
*Scopolia japonica *	Solanaceae	Root	100	−18.32 ± 1.18	>100
*Chloranthus japonicus *	Chloranthaceae	Whole plant	100	31.04 ± 2.37	>100
*Barbarea orthoceras *	Cruciferae	Whole plant	100	−27.85 ± 2.32	>100
*Caulophyllum robustum *	Berberidaceae	Stem, leaf	100	−4.46 ± 3.06	>100
*Caulophyllum robustum *	Berberidaceae	Root	100	−23.10 ± 6.27	>100
*Carduus crispus *	Compositae	Stem, leaf	100	30.13 ± 3.47	>100
*Carduus crispus *	Compositae	Flower	100	44.24 ± 2.47	>100
*Styrax japonica *	Styracaceae	Flower	100	31.62 ± 4.47	>100
*Cornus controversa *	Cornaceae	Branch, leaf	100	39.65 ± 5.62	>100
*Cornus controversa *	Cornaceae	Flower	100	40.45 ± 0.66	>100
*Magnolia sieboldii *	Magnoliaceae	Branch, leaf	100	4.84 ± 5.72	>100
*Magnolia sieboldii *	Magnoliaceae	Flower	100	−7.03 ± 8.14	>100
*Prunus persica *	Rosaceae	Fruit	100	27.35 ± 1.98	>100
*Rhamnus yoshinoi *	Rhamnaceae	Branch, leaf	100	43.98 ± 7.76	>100
*Erigeron annuus *	Compositae	Whole plant	100	26.14 ± 0.86	>100
*Styrax japonica *	Styracaceae	Branch, leaf	100	27.88 ± 0.97	>100
*Quercus aliena *	Fagaceae	Branch, leaf	100	45.95 ± 1.73	>100
*Callicarpa japonica *	Verbenaceae	Branch, leaf	100	11.36 ± 2.56	>100
*Ligustrum obtusifolium *	Oleaceae	Branch, leaf	100	4.18 ± 1.41	>100
*Lindera obtusiloba *	Lauraceae	Branch, leaf	100	41.98 ± 1.40	>100
*Lespedeza bicolor *	Leguminosae	Branch, leaf	100	47.02 ± 2.78	>100
*Carpinus laxiflora *	Betulaceae	Branch, leaf	100	39.49 ± 5.62	>100
*Machilus thunbergii *	Lauraceae	Bark	100	36.58 ± 3.17	>100
*Hedera rhombea *	Araliaceae	Whole plant	100	29.92 ± 0.78	>100
*Arenaria serpyllifolia *	Caryophyllaceae	Whole plant	100	13.09 ± 1.54	>100
*Paulownia coreana *	Paulowniaceae	Flower	100	35.25 ± 1.77	>100
*Thlaspi arvense *	Brassicaceae	Whole plant	100	0.32 ± 0.92	>100
*Vicia villosa *	Leguminosae	Whole plant	100	28.71 ± 1.94	>100
*Descurainia pinnata *	Brassicaceae	Whole plant	100	7.88 ± 1.21	>100
*Ribes fasciculatum *	Saxifragaceae	Branch, leaf, fruit	100	33.67 ± 2.10	>100
*Corydalis speciosa *	Fumariaceae	Whole plant	100	9.30 ± 3.47	>100
*Clematis fusca *	Ranunculaceae	Whole plant	100	−1.24 ± 5.89	>100
*Deutzia parviflora *	Saxifragaceae	Branch, leaf, stem, flower	100	34.77 ± 3.21	>100
*Rosa multiflora *	Rosaceae	Branch, leaf, stem, flower	100	42.42 ± 0.26	>100
*Parthenocissus tricuspidata *	Vitaceae	Leaf, stem	100	48.73 ± 1.62	>100
*Chelidonium majus *	Papaveraceae	Whole plant	100	10.93 ± 1.55	>100
*Platycarya stobilacea *	Juglandaceae	Leaf	100	47.97 ± 1.14	>100
*Platycarya stobilacea *	Juglandaceae	Flower	100	46.63 ± 0.54	>100
*Carpinus cordata *	Betulaceae	Leaf	100	45.84 ± 1.30	>100
*Celtis sinensis *	Ulmaceae	Leaf	100	40.23 ± 0.47	>100
*Orixa japonica *	Rutaceae	Leaf	100	−0.19 ± 2.17	>100
*Orixa japonica *	Rutaceae	Branch, stem	100	15.79 ± 3.07	>100
*Orixa japonica *	Rutaceae	Fruit	100	25.89 ± 5.92	>100
*Picrasma quassioides *	Simaroubaceae	Leaf	100	40.51 ± 0.74	>100
*Picrasma quassioides *	Simaroubaceae	Fruit	100	25.21 ± 2.08	>100
*Tilia mandshurica *	Tiliaceae	Leaf	100	42.08 ± 1.27	>100
*Aralia cordata *	Araliaceae	Whole plant	100	32.27 ± 4.39	>100
*Viburnum sargentii *	Caprifoliaceae	Branch, leaf	100	27.00 ± 1.59	>100
*Polygonatum odoratum *	Liliaceae	Root	100	36.72 ± 0.40	>100
*Astragalus membranaceus *	Leguminosae	Root	100	−4.26 ± 0.91	>100
*Pleuropterus multiflorus *	Polygonaceae	Root	100	−17.48 ± 1.88	>100
*Torilis japonica *	Umbelliferae	Fruit	100	−20.02 ± 4.86	>100
*Phaseolus angularis *	Leguminosae	Fruit	100	−58.89 ± 0.70	>100
*Phaseolus radiates *	Leguminosae	Fruit	100	−98.96 ± 9.06	>100
*Artemisia scoparia *	Compositae	Aerial part	100	−21.76 ± 3.22	>100
*Solanum tuberosum *	Solanaceae	Tuber	100	−38.90 ± 4.60	>100
*Brassica juncea *	Cruciferae	Leaf	100	−34.85 ± 7.98	>100
*Arctium lappa *	Compositae	Root	100	−38.38 ± 7.90	>100
*Cucumis sativus *	Cucurbitaceae	Fruit	100	−138.86 ± 0.64	>100
*Diospyros kaki *	Ebenaceae	Fruit	100	−136.26 ± 6.37	>100
*Artemisia princeps *	Compositae	Aerial part	100	12.82 ± 2.47	>100
			0.0005	5.53 ± 3.21	
Orlistat (positive control)			0.005	21.40 ± 10.76	0.036 (0.073 *μ*M)
			0.05	63.19 ± 7.04	

^a^Results are the mean ± SD (*n* = 3).
